# Supplementary material for: Adequate vitamin A rich food consumption and associated factors among lactating mothers visiting child immunization and post-natal clinic at health institutions in Gondar Town, Northwest Ethiopia
Source: PLoS One. 2020 Sep 21;15(9):e0239308. doi: 10.1371/journal.pone.0239308 (PMC7508326; doi:10.1371/journal.pone.0239308)
Supplement: Attachment — Submitted filename: Manuscript of vitamin A-commented.docx [file pone.0239308.s002.docx]

To: Editorial Board

**PLOS ONE**

**11 October, 2019**

Accompanying this letter you will find our research article manuscript entitled

**“Adequate vitamin A rich food consumption and associated factors among lactating mothers visiting child immunization and post-natal clinic at health institutions in Gondar Town, Northwest Ethiopia”** submitted to your prestigious journal. The research article provides information on vitamin A rich food consumption and associated factors among lactating mothers visiting child immunization and post-natal clinic at health institutions in Gondar. This article is not yet submitted for any other journal including **PLOS ONE**, thus it is new manuscript to be submitted to **PLOS ONE** for publication. We hope the manuscript will suit for publication on this prestigious journal.

With best regards,

**Corresponding Author:**

Addisalem Damtie (MPH/Nutrition)

Phone number: +251966394068

Email: [addis2010ec@gmail.com](mailto:addis2010ec@gmail.com)

Arba Minch University

**(Department of Public Health, College of Medicine and Health Sciences, Arba Minch University, P.O. Box: 21 Arba Minch, Ethiopia**

**Adequate vitamin A rich food consumption and associated factors among lactating mothers visiting child immunization and post-natal clinic at health institutions in Gondar Town, Northwest, Ethiopia**

Addisalem Damtie Aserese^1*^, Azeb Atenafu^2^, Mekonnen Sisay^2^, Muluken Bekele Sorrie^1^, Birhanu Wubale Yirdaw^2^, Martha Kassahun Zegeye³,

^1^Arbaminch University, Ethiopia

^2^University of Gondar, Ethiopia

^3^Ambo University, Ethiopia

**Authors’ email addresses**

Addisalem Damtie: [addis2010ec@gmail.com](mailto:addis2010ec@gmail.com)

Azeb Atenafu: [bemnyaz@gmail.com](mailto:bemnyaz@gmail.com)

Mekonnen Sisay: [mekudesu@gmail.com](mailto:mekudesu@gmail.com)

Muluken Bekele: [mulukenbekele51@gmail.com](mailto:mulukenbekele51@gmail.com)

Birhanu Wubale: [Birhanuwubale@gmail.com](mailto:Birhanuwubale@gmail.com)

Martha Kassahun: [martykassahun@gmail.com](mailto:martykassahun@gmail.com)

**Corresponding author***

Addisalem Damtie (MPH/Nutrition)

Phone number: +251966394068

Email: [addis2010ec@gmail.com](mailto:addis2010ec@gmail.com)

Arba Minch University

**(Department of Public Health, College of Medicine and Health Sciences, Arba Minch University, P.O. Box: 21 Arba Minch, Ethiopia**

**Abstract**

**Background**: Vitamin A deficiency is highly prevalent in low-income countries and is the major public health problem worldwide. Lactating mothers are the most vulnerable population group to vitamin A deficiency. Despite this, there is limited study on vitamin A-rich food consumption by lactating mothers in Ethiopia. Therefore, this study aimed to assess adequate vitamin A rich food consumption and associated factors among lactating mothers visiting child immunization and postnatal care centers in health institutions of Gondar Town.

**Methods:** Institution-based cross-sectional study design was employed at health institution in Gondar Town from February to March 2019. About 631 study participants were included in the study. Simple random sampling followed by systematic sampling technique was used to select participants. The data were collected using HKI-FFQ, entered using Epi-Info 7 statistical software and exported to STATA version 14 for analysis. A multivariable logistic regression analysis was used to identify factors associated with the outcome variable and variables with p-value <0.05 were considered as statistically significant.

**Result:** A total of 624 lactating mothers participated in the study giving a response rate of 98.89%. The study shows adequate consumption of vitamin A-rich food was 38.94% (95% CI: 35%- 43%). Predictors such as attending college diploma and above (AOR= 2.26, 95% CI; 1.02- 4.99) , having household family size ≤ 3 (AOR= 4.04, 95% CI; 1.60-10.17), being in higher economic class (AOR= 1.93 ,95% CI; 1.18-3.14), having dietary diversity score of ≥ 5 (AOR=1.59, 95% CI; 1.09- 2.32) and meal frequency of ≥ 4 (AOR= 1.64, 95% CI; 1.09- 2.32) were statistically significant.

**Conclusion and recommendation:** The majority of respondents had inadequate consumption of foods rich in vitamin A. Educational status, family size, wealth index, dietary diversity and meal frequency were found to be factors that affect adequate consumption of vitamin A-rich foods. Encouragement and more education for lactating mothers to consume foods rich in vitamin A are crucial.

## **Keywords:** Vitamin A, adequate consumption, predictors, lactating mothers, HKI-FFQ, Gondar, Ethiopia

## **Background**

Vitamin A is an essential fat-soluble vitamin required in a small amount for human beings for the better performance of different systems of our body including vision, reproduction, growth, and development, immunity and integration of epithelium (1,2).

Two forms of vitamin A are available in foods namely preformed vitamin A (retinol) which is found in animal products such as human milk, glandular meats, liver and fish liver oils, egg yolk, whole milk, and other dairy products and provitamin A (carotenoids) that is found in plant sources like green leafy vegetables (e.g. spinach, kale), yellow vegetables (e.g. pumpkins, squash, and carrots), and yellow and orange non-citrus fruits (e.g. mangoes, apricots and papayas (3). The recommended daily allowance (RDA) of vitamin A for lactating mothers is 1300μg/d (4).

Lactating mothers are the most vulnerable population to vitamin A deficiency because they need a high amount to provide enough amount of micronutrients during lactation and to replace the lost occur through breastfeeding (5).

Vitamin A deficiency is found to be the major public health problem in different countries especially among low-income countries (2,6,7)**.** It is the leading cause of morbidity and mortality for nutritionally vulnerable population segments including lactating mothers (8)**.**

It has been given little attention for a long time for the problem of vitamin A deficiency among breastfeeding mothers as compared to preschool children having the characteristics of a rare clinical symptoms of xeropthalmia in women (9). However, it should be noted that lactating mothers are at risk of vitamin A deficiency due to the high requirement of the vitamin as a result of breastfeeding (10).

Epidemiological studies revealed a high prevalence of low levels of serum retinol in lactating mothers in different Asian countries including 37% in Bangladesh (11), 13% in Bhaktapur, Nepal (12) and 18% in Indonesia (13). Moreover, a high magnitude of vitamin A deficiency was demonstrated by different African countries. About 40% of lactating mothers were found to be vitamin A deficient in Makhaza, Zimbabwe (14), 38% mothers in Zambia (15) and 10% women were severely deficient in Kenya (16).

In Ethiopia, researches showed that vitamin A deficiency remains public health concern and found a high prevalence of national bitot’s spot with the highest level being in the Amhara region (17–19). According to the Ethiopian national micronutrient survey report, the national prevalence of vitamin A deficiency among women of the reproductive age group was found to be 3.4% (20).

Several causes were identified for the development of vitamin A deficiency. Inadequate consumption of vitamin A rich foods, socio-demographic and economic status, duration of lactation (prolonged), amount of body fat and hemoglobin concentration were some of them (6,13,14,16,21).

Vitamin A deficiency has a wide range of consequences. Ocular manifestations (xerophthalmia), mortality, failure to thrive, poor reproduction are most commonly manifested problems among vitamin A deficient group (22). In addition to the above consequences, vitamin A deficiency has an intergenerational effect. Children with VAD are usually born from mothers with vitamin A deficiency who cannot produce enough amount of vitamin A in breast milk which results in vitamin A deficiency to the child and puts the next generation at risk of vitamin A deficiency (9).

Attempts were made to prevent and control vitamin A deficiency including promoting vitamin A rich food consumption, providing vitamin A supplementation to lactating mothers and children, treatment of conditions and disease with vitamin A and fortification of vitamin A with cooking oil (23).

Ethiopia launched the revised national nutrition program which included strengthen of nutrition- sensitive intervention that mainly focused on promoting home gardening, community horticulture production, strengthen fruit and vegetable, promoting urban gardening, increase access and utilization of animal source foods as the part of strategic objective and this has great contribution to prevent vitamin A deficiency (24). However, little is known regarding the consumption of vitamin A rich food among lactating mothers. Hence, the present study was aimed at assessing the consumption of vitamin A rich foods and associated factors by lactating mothers.

# Methods

## **Study design and setting**

Institution-based cross-sectional study was conducted from February to March 2019 at health institutions in Gondar Town. Gondar Town is located in the North Gondar Zone of the Amhara Region and is 750kms away from Addis Ababa, the capital city of Ethiopia. It is North of Tana Lake on the lesser Angereb River and Southwest of the Semien Mountains. It has a latitude and longitude of 12°36′N and 37°28′E respectively with an elevation of 2133 meters above sea level. According to The Gondar Town health office report currently, the total population of the city is about 338,646 of which, 79,853 and 45,853 are women of reproductive age group and under five children respectively. It is estimated that there are 11,412 pregnant and lactating mothers in the Town. There are 8 health centers and one comprehensive specialized referral hospital. The major food products are maize, sorghum, teff, bean, and other legumes. potato, spinach, lettuce, cabbage, kale, pumpkin, and fruits like mango, avocado, banana,and orange are also available.

## **Sample size calculation and sampling procedure**

All lactating mothers visiting child immunization and postnatal centers at health institutions in Gondar Town were considered as source population while those mothers in the selected health facilities were considered as the study population. Lactating mothers visiting child immunization (EPI) and postnatal care (PNC) centers in selected health facilities during the study period in Gondar Town were included in the study.

The sample size for the intended study was calculated based on single population proportion assumptions. Assuming 95% confidence level, 50% prevalence of consumption of vitamin A rich food, a margin of error 5%, design effect of 2 and 10% of non-response rate, after applying correction formula, the final sample size was 631.

To select the study subjects, simple random followed by systematic sampling was employed. From 9 health facilities, 5 health institutions (Gondar university comprehensive specialized referral hospital, Maraki Health Center (HC), Poly HC, Azezo HC, and Teda HC), were selected by simple random sampling. By allocating sample proportionally, 179 mothers from Gondar University Hospital, 124 mothers from Gondar Poly Health Center, 172 mothers from Maraki Health Center, 84 mothers from Azezo Health Center and 72 mothers from Teda Health Center were selected. The first participant was selected using the lottery method and every other mother was included until the desired sample size was achieved.

## **Operational definitions**

**Adequate Vitamin A rich foods consumed**: Consumption of animal sources of vitamin A for > 4 days per week, or consumption of weighted source (total consumption of animal and vegetable sources of vitamin A) for > 6 days per week (27).

**Minimum women dietary diversity**: Consumption of at least 5 food groups from 10 food groups (29).

**Poor nutritional knowledge**: Individuals with scores 0-3 from 9 points were classified as having poor nutrition knowledge.

**Fair nutritional knowledge**: Those with scores 4-6 were considered as having fair nutritional knowledge.

**Adequate nutritional knowledge**: Those with scores of 7-9 were classified as having adequate nutritional knowledge (30).

**Data collection tool and procedure**

The data were collected via interview using structured and pretested questionnaire developed from different sources mainly Hellen Keller International Food Frequency Questionnaire after adapting in to the local context for measuring consumption of vitamin A-rich foods (27), Food and Agriculture Organization (FAO) to measure woman’s dietary diversity (29), and other literatures. Consumption of vitamin A-rich food was assessed with food items listed in the Hellen Keller guideline. Those food items included for the analysis of vitamin A-rich food consumption includes plant source foods (dark green leafy vegetables (DGLVs), carrot, pumpkin, mango, papaya, palm oil) and animal sources (egg, fish, liver, butter).

The maternal dietary diversity score was collected and calculated as the sum of the number of different food groups consumed by the mother in the 24hr preceding the survey. A total of ten food groups were considered in this study which includes; grains, pulses, beans and lentils, nuts and seeds, milk and dairy products, meat and fish, egg, dark green leafy vegetables, other vitamins A-rich fruits and vegetables, other vegetables and other fruits.

The principal component analysis was used to assess the household wealth status of the respondents for urban and rural residents by considering their household assets and properties, and it was categorized into lowest, middle and highest tertiles. Ten data collectors who have BSc degree in nursing and 5 supervisors with master’s degree participated in the data collection procedure.

#### Data quality assurance

The consistency of the questionnaire was maintained by translating the English version to Amharic and then back to English. Before the actual data collection day, training was given for data collectors and supervisors. The pretest was conducted among 31 respondents at Ginbot 20 health center and necessary modification was made accordingly. The accuracy and completeness of the collected data were checked daily by the principal investigator. Moreover, the data was checked by inspection and crosschecking entered data with the questionnaire, and running frequency.

## **Data processing and analysis**

Data was entered using Epi-info version 7 statistical software and exported to Stata version 14 for analysis. Descriptive and summary statistics were performed to describe the study population and were presented using tables and graphs. Wealth index was analyzed by using principal component analysis. Factors associated with the consumption of vitamin A-rich food sources were identified by using a binary logistic regression model after checking the necessary assumptions of the model. Variables that full filled the Chi-square assumptions with a p-value of < 0.2 in the bivariable analysis were considered for multivariable logistic regression analysis. Those variables with p-value < 0.05 in multivariable logistic regression analysis were considered as statistically significant. Both crude and adjusted odds ratios with the corresponding 95% confidence interval were calculated to measure the presence and strength of the association between adequate vitamin A consumption and contributing factors. Hosmer and Lemeshow goodness of fit test was conducted to test the model fitness and the model was adequate (p-value = 0.872). Multicollinearity was checked by using variance inflation factor (VIF) and no multicollinearity was found (VIF < 5).

# Results

## **Socio-demographic and economic characteristics**

A total of 624 lactating mothers have participated in the study giving a response rate of 98.89%. The median (median ± IQR) age of the respondents was 27± 6 years. The majority (84.13%) of mothers were urban residents. About 22.9% of mothers had an educational level of diplomas and above. Half of the study subjects had a household family size of ≤ 3 and around 33.2% of respondents were within the higher class in terms of economic status (Table 1).

Table 1: Socio-demographic and economic characteristics of lactating mothers visiting EPI and PNC at health institutions in Gondar Town, Northwest Ethiopia, 2019 (n= 624)

| **Variable Category** | | **Frequency** | **Percent (%)** |
| --- | --- | --- | --- |
| **Residence** | Rural | 99 | 15.9 |
|  | Urban | 525 | 84.1 |
| **Age group** | ≤ 24 years | 184 | 29.5 |
|  | 25-34 years | 356 | 57.1 |
|  | ≥ 35 years | 84 | 13.5 |
| **Religion** | Orthodox | 531 | 85.1 |
|  | Muslim | 87 | 13.9 |
|  | Others (protestant, catholic) | 6 | 1.0 |
| **Marital status** | Single | 28 | 4.5 |
|  | Married | 552 | 88.5 |
|  | Cohabiting | 26 | 4.2 |
|  | Others (widowed, divorced) | 18 | 2.9 |
| **Educational status** | Unable to read and write | 109 | 17.5 |
|  | Able to read and write | 115 | 18.4 |
|  | Primary school | 118 | 18.9 |
|  | Secondary school | 139 | 22.3 |
|  | Diploma and above | 143 | 22.9 |
| **Occupation** | Government employee | 109 | 17.5 |
|  | NGO employee | 15 | 2.4 |
|  | Self-employed | 124 | 19.9 |
|  | Housewife | 356 | 57.1 |
|  | Others (student, daily laborer) | 20 | 3.2 |
| **Family size** | 1-3 | 312 | 50.0 |
|  | 4-6 | 266 | 42.6 |
|  | >6 | 46 | 7.4 |
| **Wealth index** | Lower class | 208 | 33.3 |
|  | Middle class | 209 | 33.5 |
|  | Higher class | 207 | 33.2 |

### Maternal dietary diversity

From the ten food groups used for assessment of maternal dietary diversity, grains (75.48%) and legumes (62.07%) were highly consumed within 24 hours preceding the survey. The proportion of mothers who consumed ≥ 5 food groups within 24 hours preceding the survey was 43.75% (Table 2).

Table 2: Food groups consumed 24 hours before the survey by lactating mothers visiting EPI and PNC at health institutions in Gondar Town, Northwest Ethiopia, 2019 (n=624)

| **Food items** | **Frequency** | **Percent (%)** |
| --- | --- | --- |
| Grains | 471 | 75.48 |
| Pulses, beans, peas and lentils | 387 | 62.07 |
| Nuts and seeds | 115 | 18.43 |
| Milk and milk products | 369 | 59.13 |
| Meat, poultry and fish | 373 | 59.78 |
| Egg | 289 | 46.31 |
| Dark green leafy vegetables | 279 | 44.71 |
| Other vitamin A-rich fruits and Vegetables | 272 | 43.59 |
| Other vegetables | 187 | 29.97 |
| Other fruits | 212 | 33.97 |
| Dietary Diversity Score |  |  |
| < 5 food groups  ≥ 5 food groups | 351  273 | 56.3  43.7 |

## **Health service and nutritional advice related characteristics**

The majority (92.8%) of the participants gave birth to their last child at the institution. About 66.7% of mothers had ANC visits 4 times and above during their last pregnancy (Table- 3).

Table- 3: Health service-related characteristics of lactating mothers visiting EPI and PNC at health institutions in Gondar Town, Northwest Ethiopia, 2019 (n=624)

| **Variable** | **Frequency** | **Percent (%)** |
| --- | --- | --- |
| **Place of delivery** |  |  |
| Institution | 579 | 92.8 |
| Home | 45 | 7.2 |
| **ANC visit** |  |  |
| < 4 | 208 | 33.3 |
| ≥ 4 | 416 | 66.7 |
| **Nutritional counselling to eat more frequently during lactation** |  |  |
| Yes | 495 | 79.3 |
| No | 129 | 20.7 |
| **Nutritional counselling to eat animal sources frequently during lactation** |  |  |
| Yes | 472 | 75.6 |
| No | 152 | 24.4 |
| **Sources of information to eat fruit and vegetables frequently during lactation** |  |  |
| Yes | 460 | 73.7 |
| No | 164 | 26.3 |

**Source of nutritional advice for lactating mothers**

The majority of study participants received information on eating frequently during lactation from health professionals (Table 4).

Table- 4: sources of nutritional advice or counselling for lactating mothers visiting EPI and PNC at health institutions in Gondar Town, Northwest Ethiopia, 2019

| **Source of information** | **Frequency** | **Percent (%)** |
| --- | --- | --- |
| **To eat more frequently during lactation (n=495)**  Health professional |  |  |
| Yes | 425 | 85.9 |
| No | 70 | 14.1 |
| Health extension workers |  |  |
| Yes | 31 | 6.3 |
| No | 464 | 93.7 |
| Family /friends |  |  |
| Yes | 59 | 11.9 |
| No | 436 | 90.5 |
| Mass media |  |  |
| Yes | 4 | 0.8 |
| No | 491 | 99.2 |
| **To eat animal sources frequently during lactation (n=472)** |  |  |
| Health professional |  |  |
| Yes | 398 | 84.3 |
| No | 74 | 15.7 |
| Health extension workers |  |  |
| Yes | 26 | 5.5 |
| No | 446 | 94.5 |
| Family /friends |  |  |
| Yes | 51 | 10.8 |
| No | 421 | 89.2 |
| Mass media |  |  |
| Yes | 18 | 3.8 |
| No | 454 | 96.2 |
| **To eat fruit and vegetables frequently during lactation (n=460)** |  |  |
| Health professional |  |  |
| Yes | 385 | 83.3 |
| No | 77 | 16.7 |
| Health extension workers |  |  |
| Yes | 33 | 7.2 |
| No | 427 | 92.8 |
| Family /friends |  |  |
| Yes | 46 | 10 |
| No | 414 | 90 |
| Mass media |  |  |
| Yes | 28 | 6.1 |
| No | 432 | 93.9 |

## **Nutritional knowledge**

Nine questions were used to assess the nutritional knowledge of the respondents. The proportion of adequate nutritional knowledge was found to be 49.8% (Table 5).

Table 5: Nutritional knowledge of lactating mothers visiting EPI and PNC at health institutions in Gondar Town, Northwest Ethiopia, 2019(n= 624).

| **Question** | **Frequency** | **Percent (%)** |
| --- | --- | --- |
| **Meaning of nutrition** |  |  |
| Related to the importance of food to the body | 436 | 69.9 |
| I don’t know | 188 | 30.1 |
| **Reason for eating** |  |  |
| For growth and development | 402 | 64.4 |
| To satisfy hunger | 222 | 35.6 |
| **Description of a balanced diet** |  |  |
| Diet containing an appropriate amount of each nutrient | 390 | 62.5 |
| Diet containing all the necessary nutrients | 234 | 37.5 |
| **Presence of disease due to inadequate intake of foods** |  |  |
| Yes | 565 | 90.5 |
| No | 59 | 9.5 |
| **Energy giving foods** |  |  |
| Starchy foods | 292 | 46.8 |
| Fruits and vegetables | 332 | 53.2 |
| **Bodybuilding foods** |  |  |
| Animal foods and legumes | 412 | 66.0 |
| Fruits, fats, and oils | 212 | 34.0 |
| **Protective foods** |  |  |
| Fruits and vegetables | 345 | 55.3 |
| Starchy roots, plantains, and oils | 279 | 44.7 |
| **Importance of snack for HIV infected person** |  |  |
| Yes | 530 | 84.9 |
| No | 94 | 15.1 |
| **Importance of exercise to human** |  |  |
| Yes | 600 | 96.2 |
| No  **Nutritional knowledge**  Poor  Fair  Adequate | 24  64  249  311 | 3.8  10.3  39.9  49.8 |

## **Vitamin A rich food consumption**

From the animal sources of vitamin A, Meat was consumed by 92.3% mothers with in the last week before the survey. Next to meat, milk was the most highly consumed animal source (69.72%). Among plant sources, palm oil (88.8%) and Dark green leafy vegetables (67.15%) were consumed relatively higher than other plant sources. Adequate consumption of vitamin A-rich food was 38.9% (95% CI: 35%- 43%) (Table 6).

Table 6: Vitamin A rich food groups consumed at least once during last week by lactating mothers visiting EPI and PNC at health institutions in Gondar Town, Northwest Ethiopia, 2019 (n=624)

| **Food groups** | **Frequency** | **Percent (%)** |
| --- | --- | --- |
| Meat | 576 | 92.3 |
| Milk | 435 | 69.7 |
| Egg | 378 | 60.6 |
| Chicken | 445 | 71.3 |
| Fish | 49 | 7.9 |
| Liver | 111 | 17.8 |
| Butter | 394 | 63.1 |
| Rice | 317 | 50.8 |
| Hot peppers | 458 | 73.4 |
| DGLVs | 419 | 67.2 |
| Carrot | 226 | 36.2 |
| Mango | 189 | 30.3 |
| Pumpkin | 95 | 15.2 |
| Papaya | 132 | 21.2 |
| Spinach | 326 | 52.2 |
| Peanut | 271 | 43.4 |
| Sweet potato | 128 | 20.5 |
| Lentil or others legumes | 544 | 87.2 |
| Palm oil | 613 | 98.2 |
| Apricot | 121 | 19.4 |

## **Factors associated with adequate consumption of vitamin A-rich food sources**

The association between adequate consumption of vitamin A-rich foods and predictors was analyzed using binary logistic regression. All factors which fulfilled the chi-square assumptions and p-value <0.2 in univariable analysis were considered to multivariable logistic regression model. Hence, residence, occupational status, educational status, household family size, wealth index, dietary diversity, meal frequency, place of delivery, ANC visit, nutritional counseling to eat more frequently during lactation, nutritional counseling to eat animal sources frequently during lactation, nutritional counseling to eat fruit and vegetables frequently during lactation and nutritional knowledge were included in to multi-variable analysis and educational status, household family size, wealth index, dietary diversity, and meal frequency were found to be statistically significant at p-value <0.05 and were considered to be the determinants for the consumption of vitamin A-rich food sources.

The odds of eating adequate vitamin A-rich foods among mothers whose educational level is college and above was 2.28 times higher as compared to those unable to read and write (AOR= 2.26, 95% CI; 1.02- 4.99). Those mothers with household family ≤ 3 had adequate consumption 4 times higher than those with family size more than 6 (AOR= 4.04, 95% CI; 1.60 - 10.17). Household economic status found to be a significant predictor of adequate consumption of vitamin A food sources. The likelihood of consumption was 1.93 times higher for mothers who were in the higher economic class (AOR= 1.93, 95% CI; 1.18-3.14) as compared to those who belong to lower economic class. Furthermore, the odds of adequate consumption among mothers who had a dietary diversity score of ≥ 5 were 1.59 times higher than their counterparts (AOR=1.59, 95% CI; 1.09- 2.32). The consumption of vitamin A-rich food was found to be 1.61 times higher for those mothers who had > 3 meals per day as compared with those who had smaller meal frequency (AOR= 1.61, 95%CI; 1.09- 2.32) (Table 7).

Table 7: Bivariable and multivariable logistic regression model predicting the likelihood of adequate consumption of vitamin A rich food sources by lactating mothers visiting EPI and PNC at health facilities in Gondar Town, Northwest Ethiopia, 2019

| Variable | Vitamin A rich food consumption | | | COR (95%CI) | AOR (95%CI) |
| --- | --- | --- | --- | --- | --- |
|  | Adequate | Inadequate | |  |  |
| **Residence** |  | |  |  |  |
| Rural | 27 | | 72 | 1 | 1 |
| Urban | 216 | | 309 | 1.86 (1.16- 2.99) | 0.79(0.41 – 1.51) |
| **Occupation** |  | |  |  |  |
| Housewife | 112 | | 244 | 1 | 1 |
| Government employee | 62 | | 47 | 2.87 (1.85- 4.46) | 0.92 (0.48- 1.77) |
| NGO employed | 7 | | 8 | 1.91 (0.67- 5.39) | 0.86 (0.25- 2.95) |
| Self employed | 55 | | 69 | 1.74 (1.14- 2.64) | 1.22 (0.76- 1.95) |
| Others | 7 | | 13 | 1.17 (0.46- 3.02) | 0. 92 (0.32- 2.63) |
| **Educational status** |  | |  |  |  |
| Unable to read and write | 30 | | 79 | 1 | 1 |
| Able to read and write | 33 | | 82 | 1.06 (0.59-1.90) | 0.73 (0.37-1.42) |
| Primary school | 40 | | 78 | 1.35 (0.77-2.38) | 0.88 (0.45-1.73) |
| Secondary school | 48 | | 91 | 1.39 (0.80-2.40) | 0.75 (0.38-1.49) |
| College and above | 92 | | 51 | 4.75 (2.76-8.17) | 2.26 (1.02- 4.99) ⃰ |
| **Family size** |  | |  |  |  |
| >6 | 7 | | 39 | 1 | 1 |
| 4-6 | 107 | | 159 | 3.94 (1.70-9.10) | 3.06 (1.03 -7.64) ⃰ |
| 1-3 | 129 | | 183 | 3.75 (1.62-8.69) | 4.04 (1.60-10.17) ⃰ |
| **Wealth index** |  | |  |  |  |
| Lower class | 55 | | 153 | 1 | 1 |
| Middle class | 74 | | 135 | 1.52 (1.00-2.31) | 1.11 (0.69 -1.76) |
| Upper class | 114 | | 93 | 3.41 (2.26-5.15) | 1.93 (1.18 - 3.14) ⃰ |
| **Dietary diversity** |  | |  |  |  |
| < 5 food groups | 114 | | 237 | 1 | 1 |
| ≥ 5 food groups | 129 | | 144 | 1.86 (1.34-2.58) | 1.59 (1.09- 2.32) ⃰ |
| **Meal frequency** |  | |  |  |  |
| ≤ 3 times | 129 | | 280 | 1 | 1 |
| > 3 times | 114 | | 101 | 2.45 (1.74- 3.44) | 1.61 (1.09- 2.32) ⃰ |
| **Place of delivery** |  | |  |  |  |
| Home | 6 | | 39 | 1 | 1 |
| Institution | 237 | | 342 | 4.50 (1.88-10.81) | 2.03(0.77- 5.31) |
| **ANC visit** |  | |  |  |  |
| < 4 visits | 55 | | 153 | 1 | 1 |
| ≥ 4 visits | 188 | | 228 | 2.29 (1.59 -3.29) | 1.36 (0.87-2.12) |
| **Nutritional counseling to eat frequently during lactation** |  | |  |  |  |
| No | 30 | | 99 | 1 | 1 |
| Yes | 213 | | 282 | 2.49 (1.59- 3.89) | 0.91 (0.44- 1.87) |
| **Nutritional counseling to eat animal during lactation** |  | |  |  |  |
| No | 36 | | 116 | 1 | 1 |
| Yes | 207 | | 265 | 2.52 (1.66- 3.81) | 1.01 (0.49- 2,04) |
| **Nutritional counseling to eat fruit and vegetables during lactation** |  | |  |  |  |
| No | 37 | | 127 | 1 | 1 |
| Yes | 206 | | 254 | 2.78 (1.85- 4.19) | 1.46 (0.77- 2.76) |
| **Nutritional knowledge** |  | |  |  |  |
| Poor | 15 | | 49 | 1 | 1 |
| Fair | 79 | | 170 | 1.52 (0.80- 2.87) | 1.08 (0.51- 2.29) |
| Adequate | 149 | | 162 | 3.00 (1.62- 5.58) | 1.71 (0.79- 3.68) |
| *= p-value <0.05, COR= crude odds ratio and AOR=adjusted odds ratio | | | | | |

# Discussion

Consumption of adequate vitamin A rich foods was found to be 38.9% (95% CI: 35%- 43%). This finding was higher than a study done in Sodo Zuria District which found that only 0.5% of preschool children had consumed animal source foods of vitamin A >4 times and 2 % were consumed a weighted source of VitaminA- rich foods > 6 times per week (31). The possible explanation for this huge difference might be; the difference in the study setting this study was institution based which enables the mothers to gain some insight about vitamin A rich foods relative to their counterparts. It can be also due to differences in the study population; giving that the study subjects were mothers in the present study (adult), they are less likely to be reluctant to eat foods as compared to children. Moreover, the difference may be because the majority (84.1%) study subjects of the current study were urban residents and most of them had educational level college and above and it was found that the consumption was higher among those mothers with high educational levels. Furthermore, the time gap between the two studies which is 8 solid years, there may be some improvements such as health service seeking behavior.

The finding was also greater than a study done in Kachabira District, which reported that 34.38% of children have consumed foods rich in vitamin A ≥ 3 times within a week (26). The discrepancy can be due to differences in the study setting, study population and period. It was also observed that the majority of mothers in the mentioned study had elementary schooling and had no education which was not the case in the current study.

Additionally, the finding was higher than a research done in Damot Sore District, which revealed only 12.5% of pregnant mothers had consumed either animal or plant source ≥3 times per week (25). The difference can be reasonably explained as difference in sample size which was reported to be 104 for the mentioned study which may lack power causing difficulties in determining the true effect, difference in study setting, physiological state (being pregnant may reduce intake by decreasing appetite) and time gap between the studies (10 years) may create variation.

Moreover, the finding was also above the finding obtained from rural Kenya which found 13.5% of mothers consumed animal sources ≥ 3 days per week. The variation might be due to the small sample size used by the mentioned study (62 mother-child pairs) which may cause a reduction of power. It may be also due to the difference in the study setting and time gap (20 years).

A higher finding was obtained as compared to a study done in India that found 20% of adequate vitamin A rich food intake (33). The discrepancy may be justified in terms of the difference in the study setting, study population, the residence of the study subjects and time gap between the current study and the mentioned study.

Furthermore, the present study had higher findings than done in Bangladesh which found 13% of lactating mothers had intakes of vitamin A above the recommended dietary allowance (RDA) (11). The possible explanation for this difference can be the difference in economic status. The study subjects Bangladesh were poor urban mothers and economic status was found to be a major determinant for the consumption of foods rich in vitamin A.

High level of adequate vitamin A rich food consumption was demonstrated among mothers with an educational status of college and above relative to those who are unable to read and write. This was supported by a study done in Nepal which showed that there was a positive relationship between adequate micronutrient intake and the mother’s educational status (5). This might be because mothers who attended college and above are more likely to get more information about vitamin A food sources and the consequence of inadequate eating.

Household family size was found to be strongly associated with adequate consumption of vitamin A-rich foods and mothers with household family size ≤ 3 had adequate consumption 4 times higher than those with > 6 family size. As more household members are available, it may be difficult to provide adequate foods to large family size and mothers may hesitate to feed themselves rather they may care about other members. They may be also busy with preparing foods for household members.

The likelihood of adequate consumption was 1.93 times higher for mothers who were higher in economic class as compared to the lower class. The possible explanation can be individuals with the good economic standards will have access and can afford foods especially animal source foods than their counterparts.

The odds of adequate consumption of vitamin A among mothers with a dietary diversity score of ≥ 5 were 1.59 higher than their counterparts. This can be supported by the study in Nepal which found a positive relation between micronutrient consumption and dietary diversity (5). According to WHO guideline, a child who consumed foods from at least 4 food groups on a preceding day has a high possibility of consumption at least one animal-source food and at least one fruit or vegetable on that day, in addition to a staple food such as grain in most populations (34).

Furthermore, adequate consumption of vitamin A-rich food was associated with the number of meals within a day. Those mothers who had > 3 meals per day were 1.61 times more likely to consume adequate vitamin A-rich foods as compared to those who had ≤ 3 meals. This may be because as the number of meals increases it is more likely to increase the variety of foods and food containing vitamin A.

**Limitations of the study**

The study was institution-based, it may fail to show the consumption of specific community and those who come to health facilities may have relatively good consumption since they may have some information regarding vitamin A-rich foods and possible consequences of inadequate intake. The study may be prone to recall bias and social desirability bias as it assessed the intake of food before a week.

# Conclusion

The study showed that the majority of the respondents had inadequate vitamin A rich food consumption. Attending college diploma and above, household family size of 3 and below, higher economic class, having minimum dietary diversity score and meal frequency of 4 and above were predictors for adequate consumption of vitamin A-rich food sources. Despite vitamin A rich food consumption was not a public health problem based on Hellen Keller threshold level, most of the mothers had inadequate consumption of foods rich in vitamin A.

**List of Abbreviations**

ANC =Antenatal care, AOR= Adjusted odds ratio, COR= Crude odds ratio, DGLVs= Dark green leafy vegetables, EPI= Expanded immunization program, EPI-INFO= Epidemiological Information, FAO= Food and Agriculture Organization, HKI-FFQ= Helen Keller international food frequency questionnaire, IQR= Interquartile range, NGO= Non-governmental organization, PNC= Postnatal care, RDA= Recommended daily allowance, VAD= Vitamin A Deficiency, VIF= Variance inflation factor, WHO= World Health Organization

**Declarations**

**Ethics approval and consent to participate**

Ethical clearance was obtained from the Ethical Review Board of the University of Gondar. A letter of permission was obtained from the Gondar town health office and the respective medical director of each health facility under study. The objective of the study was explained and consent was secured from study subjects. The right of participants to withdraw from the study at any time without any precondition was disclosed. Moreover, the confidentiality of information obtained was guaranteed by all data collectors and investigators by using code numbers rather than personal identifiers.

**Consent for publication**

Not applicable in this section

**Availability of data and materials**

Data will be available upon reasonable request from the corresponding author.

**Competing interest**

Authors declare that they had no conflict of interest

**Funding**

No funding was obtained for this study.

**Authors' contributions**

All authors conceived the study, participated in the design of the study and tool development, coordinate data collection, performed statistical analysis, drafted and write the final version of the manuscript. All authors have read and approved the final manuscript.

**Acknowledgements**

We are indebted to the study participants, data collectors and supervisors for their time and commitment to take part in the study.

# References

1.	Mclaren DS, Frigg M. SIGHT AND LIFE MANUAL ON VITAMIN A. 2001.

2.	Database WHO. Global prevalence of vitamin A deficiency in populations at risk. 2005;

3.	WHO/FAO. Vitamin and mineral requirements in human nutrition Second edition. WHO. 2004;

4.	Kominiarek MA, Rajan P. HHS Public Access. 2017;100(6):1199–215.

5.	Henjum S, Torheim LE, Thorne-Lyman AL, Chandyo R, Fawzi WW, Shrestha PS, et al. Low dietary diversity and micronutrient adequacy among lactating women in a peri-urban area of Nepal. Public Health Nutr. 2015;18(17):3201–10.

6.	Walters D, Wu D, Saleh N, Horton S, Laswai HS, Sciences C, et al. Factors Influencing Vitamin A Status of Lactating Mothers in Manyara and Shinyanga Regions of Tanzania. 2016;15(1):21–32.

7.	Samba C, Tchibindat F, Gourmel B, Houzé P, Malvy D. Prevalence of vitamin A deficiency in pregnant and lactating women in the republic of Congo. J Heal Popul Nutr. 2013;31(1):28–36.

8.	Greiner T. VITAMIN A : MOVING THE FOOD-BASED APPROACH FORWARD Ted Greiner Professor of Nutrition Department of Nutrition Hanyang University Seoul 133-791 South Korea. fao. 2013;1–69.

9.	Miller M, Humphrey J, Johnson E, Marinda E, Brookmeyer R, Katz J. Proceedings of the XX International Vitamin A Consultative Group Meeting Why Do Children Become Vitamin A Deficient ? 1 , 2. 2002;2867–80.

10.	Deficiency S, Rice AL, Stoltzfus RJ, Francisco A De, Chakraborty J, Kjolhede CL, et al. Community and International Nutrition Maternal Vitamin A or ␤ -Carotene Supplementation in Lactating Bangladeshi Women Benefits Mothers and Infants but Does Not Prevent. 1998;(July):356–65.

11.	Ahmed F, Azim A, Akhtaruzzaman M. Vitamin: A deficiency in poor, urban, lactating women in Bangladesh: factors influencing vitamin A status. Public Health Nutr [Internet]. 2003;6(05):447–52.

12.	Ulak M, Strand TA, Thorne-lyman AL, Henjum S, Shrestha PS, Fawzi WW. Prevalence of vitamin A deficiency among lactating mothers and infants in Bhaktapur , Nepal. :4.

13.	Dijkhuizen MA, Wieringa FT, West CE. Concurrent micronutrient deficiencies in lactating mothers and. Am J Clin Nutr. 2001;73:786–91.

14.	Ncube TN, Malaba L, Greiner T, Gebre-Medhin M. Evidence of grave vitamin A deficiency among lactating women in the semi-arid rural area of Makhaza in Zimbabwe. A population-based study. Eur J Clin Nutr. 2001;55(4):229–34.

15.	African E, Journal M. IRON AND VITAMIN A STATUS OF BREASTFEEDING MOTHERS IN ZAMBIA. 2001;(9).

16.	Ettyang GA, Van Marken Lichtenbelt WD, Oloo A, Saris WHM. Serum Retinol, Iron Status and Body Composition of Lactating Women in Nandi, Kenya. Ann Nutr Metab. 2003;47(6):276–83.

17.	Azage T, Claude M. NUTRITION COUNTRY PROFILE Acknowledgments. 2008;

18.	Demissie T, Ali A, Mekonen Y, Haider J, Umeta M. Magnitude and distribution of vitamin A deficiency in Ethiopia. Food Nutr Bull. 2010;31(2):234–41.

19.	Haidar J. Vitamin A Deficiency in Ethiopia in the last 50 Years. 2016;(January).

20.	Hailu AA. Ethiopian National Micro nutrient survey. 2017;(September 2016).

21.	WHO. World Health Organization (2009) Global prevalence of vitamin A deficiency in populations at risk 1995 – 2005: WHO global database on vitamin A deficiency. Geneva, Switzerland. WHO Press. 2009;

22.	Sommer A. vitamin A deficency and its consequences. WHO. 1995;

23.	USAID. Essential nutrition action and essential hygiene actions. 2015;(April).

24.	FDRE. National Nutrition Programme of Ethiopia. 2015;(June).

25.	Abebe, Hiwot; Abebe, Yewelsew; Loha, Eskindir; Stoecker BJ. Consumption of vitamin a rich foods and dark adaptation threshold of pregnant women at Damot Sore District, Wolayita, Southern Ethiopia. Ethiop J Heal Sci [Internet]. 2014;24(3):219–226. Available from: http://www.ncbi.nlm.nih.gov/pmc/articles/PMC4141225/

26.	Jemberu Y, Zegeye M, Singh P, Abebe H. Consumption of Vitamin A Rich Foods and Associated Factors among Children Aged 6-23 Months in Kachabira. Int J Food Sci Nutr Eng. 2017;7(1):1–10.

27.	Persson V. Vitamin A intake , status and improvement using the dietary approach. 2001.

28.	FAO. Minimum Dietary Diversity for Women- A Guide to Measurement. Minimum Dietary Diversity for Women: A Guide for Measurement. 2016. 82 p.

29.	Nti CA, Hayford J, Opare-obisaw C. Nutrition Knowledge , Diet Quality and Nutritional Status of People Living with HIV ( PLHIV ) in Ghana. 2012;2(6):219–27.

30.	Omer Seid, pragta Singh GB. Assessment of Dietary Consumption of Vitamin A by Preschool Children in Assessment of Dietary Consumption of Vitamin A by Preschool Children in Southern Ethiopia- A Cross Sectional Study. 2016;(January).

31.	Institute E public health. Ethiopia National Food Consumption Survey. 2013;

32.	Suri S, Kumar D, Das R. Dietary deficiency of vitamin A among rural children : A community-based survey using a food-frequency questionnaire. 2017;61–72.

33.	WHO. Indicators for assessing infant and young child feeding practices. WHO. 2008
