# Supplementary material for: Adequate vitamin A rich food consumption and associated factors among lactating mothers visiting child immunization and post-natal clinic at health institutions in Gondar Town, Northwest Ethiopia
Source: PLoS One. 2020 Sep 21;15(9):e0239308. doi: 10.1371/journal.pone.0239308 (PMC7508326; doi:10.1371/journal.pone.0239308)
Supplement: Attachment — Submitted filename: Response for editor and reviewers.docx [file pone.0239308.s003.docx]

Dear Editor and Reviewer

Sincerest thanks for your response and reviewers comments on our manuscript. We have modified the paper in response to the extensive and insightful editor and reviewer comments. We would be glad to respond to any further questions and comments that you may have.

Response to Editor

1. Is the manuscript presented in an intelligible fashion and written in standard English?
    We tried to amend the English according to the comment
2. Line 168: It is not clear why authors used 50% prevalence while they have previous study results. Of course those are community-based studies and may not apply for institutional-based study like this one. However authors have used them to compare the prevalence with current study. Authors may specify reason of using p-value of 50% and include lack of previous studies for comparison (if that is the case) as a limitation

- We used 50% prevalence to get the maximum sample size, since there is no study conducted among lactating mothers, we put it as a limitation

3. What is your rationale to implement a designed effect? What are your inclusion and exclusion criteria in this study?

- We used design effect of 2 to consider the loss of effectiveness by the use of cluster sampling, instead of simple random sampling.
- The inclusion criteria were lactating mothers visiting child immunization (EPI) and postnatal care (PNC) centers in selected health facilities during the study period in Gondar Town.
- The exclusion criteria were lactating mothers who were severely ill during data collection period; we didn’t get such type of respondent.
- We used median with IQR since the data is not normally distributed

4. The English language needs extensive revision (detailed suggestions can be found with the attachment).

- We have extensively done the copy edit for our language usage.

5. Line 56: Using abbreviation in abstract is not recommended. This abbreviation appeared for the first time here, and abbreviations should be written in full form when they appear for the first time

- It is modified accordingly to Helen Keller International Food Frequency Questionnaire

6. Line 134: As "VAD" appears for the first time it is good if it is written in full form. In general as this abbreviation only occurs at once in the entire manuscript, it is good to use in its full form.

- It is modified accordingly to vitamin A deficiency

7. Line 143: "strengthen fruit and vegetable" is not clear.

- This was to mean strengthen fruit and vegetable consumption, and corrected accordingly

8. Lines 150-160: citation is needed.

- Thank you for the comment, we have provided the citation

9. Line 168: It is not clear why authors used 50% prevalence while they have previous study results. Of course those are community-based studies and may not apply for institutional-based study like this one. However authors have used them to compare the prevalence with current study. Authors may specify reason of using p-value of 50% and include lack of previous studies for comparison (if that is the case) as a limitation.

- We used 50% prevalence to get the maximum sample size, since there is no study conducted among lactating mothers; we put it as a limitation for using in the discussion part

10. Lines 192-193: Authors mentioned that pretested questionnaire was developed from different sources mainly Hellen Keller International Food Frequency Questionnaire. Authors are encouraged to cite at least some of these sources.

- We have cited the references accordingly; when we say different sources it is to mean that references like Food and Agriculture Organization (FAO) to measure woman’s dietary diversity

11. Lines 193-194: Authors mentioned that the HK-FFQ was adapted to the local context. Did that mean the tool is validated? If so, the tool validation result may be cited or at least explicitly mentioned.

- For this study we didn’t do tool validation, since the HK-FFQ tool is internationally accepted tool for assessing vitamin A rich food intakes, the tool have 28 different food item questions. From the 28 food items we used 21 food items which are available in the study area and consumed by the society, the remaining 7 food items like, noodles, Amaranth leaves, sweet potato leaves, cod liver oil, coconuts, weaning food fortified with vitamin A, Margarine fortified with vitamin A were not included in the tool because they are not commonly consumed in the study area. Accordingly food frequency questionnaires can be adjusted to the local context.

12. Lines 213 to 214: Authors mentioned that the tool was pretested and necessary modifications made. What modifications were actually performed? What was the reliability of nutritional knowledge questions from the pretest result?

- The modifications were mainly editorial like language translation of food items from English to Amharic. We didn’t perform reliability test for the nutritional knowledge questions, since it is the commonly used tool for assessing the nutritional knowledge of mothers

13. A line 220 to 221 is a repetition from line 205.

- Modified accordingly

14. Lines 230 to 231: Variance inflation factor is more suitable for linear regression and authors may use standard error to check co-linearity in binary logistic regression.

- Of course standard error is also used for checking multicollinearity, in our case standard error is also less than 4, and moreover, multicollinearity is a state of very high inter-correlations or inter-associations among the independent variables. It is therefore multicollinearity can also be detected with the help of tolerance and its reciprocal, called variance inflation factor (VIF).

15. Line 332: Authors should check the reference. The WHO report cannot be cited for a study in India.

- Checked and modified accordingly

16. Line 336: Authors used both full form and abbreviation RDA twice both at line 105 and here. What is the need of abbreviating recommended dietary allowance if the full form is always written?

- Modified accordingly

17. Line 368: This statement should be rewritten in a meaningful manner.

- Modified to consumption of specific community

18. Lines 371 to 372: This statement should be reworked as assessment of intake of food before a week seems reason of social desirability. I suggest the authors to split the statement in to two.

- Noted and modified

19. Line 389: Authors may include the review protocol number.

- The protocol number for Ethical review was IPH/180/06/2011

20. Line 391: Authors may wish to disclose whether the consent obtained was written or verbal.

- The consent obtained was verbal and included in the document

21. Finally, the discussion seems straight jacketed. Authors may include additional references and suggest additional justification. Authors may also discuss factors which are not associated in the current study but were identified by previous studies. Factors which are not associated are also important

- Some amendments were made for the discussion part, the main problem was lack of literatures
