# Supplementary material for: Adequate vitamin A rich food consumption and associated factors among lactating mothers visiting child immunization and post-natal clinic at health institutions in Gondar Town, Northwest Ethiopia
Source: PLoS One. 2020 Sep 21;15(9):e0239308. doi: 10.1371/journal.pone.0239308 (PMC7508326; doi:10.1371/journal.pone.0239308)
Supplement: Attachment — Submitted filename: Response to editor and reviewers.docx [file pone.0239308.s004.docx]

Dear Editor and Reviewer

Sincerest thanks for your response and reviewers comments on our manuscript. We have modified the paper in response to the extensive and insightful editor and reviewer comments. We would be glad to respond to any further questions and comments that you may have.

Response to Editor and Reviewer

1. Abstract
   (line 54) : can authors clarify ... the authors stated that about 631 study participants were included ... and in line 61: a total of 624 of lactating women participated... can author give the reason of the loss (-7)

Seven lactating women discontinue the interview after starting due to their personal reasons

2. Background Needs to be organized… I suggest to the authors to start the first paragraph by line 111…117

Thank you for the suggestion and we have modified accordingly

3. Line 112 : not only vitamin A deficiency is the leading cause of morbidity and mortality, also zinc deficiency and both are the most leading causes…

Yes it is not the only deficiency others also have their own great contribution. But in this paragraph we tried to be specific to vitamin A. We have modified

4. Line 113 : … vulnerable population segment by for those who have inadequate intake of vitamin A from food

Modified accordingly

5. Line 114 : lactating women are the most… in addition to children under 5y old.

Modified accordingly

6. Line 115 : … amount of micronutrients… I think here you have to put enough of vit A

Modified accordingly

7. Line 118… line 122 can be deleted

We believe it will give some insight

8. Line 129 : « …remains… » is there any measures taken nationally?

There was a measure taken to prevent this problem by preparing guidelines for the prevention and control of micronutrient deficiencies at national level

9. Line 132 : … of reproductive age… what about lactating women ? is there any regional or national statistics ?

We didn’t get data which represents lactating women at regional or national level

10. Authors can reinsert here paragraph starting by line 98 before line 134

Modified accordingly

11. Line 138 to line 144 , this paragraph needs edits and rephrasing (children who born with low vit A reserves and from deficient mothers are at greater risk to develop a vit A deficiency

Modified accordingly

12. Line 202 : adapted questionnaire instead of pretested questionnaire

Modified accordingly

13. Line 206 : .. and other litteratures, can the authors be more precise

Modified accordingly

14. I suggest to authors that data quality assurance comes after data analysis

Modified accordingly

15. Line 226 running frequency ????

Modified accordingly, it was not done for this research; we removed the statement from the paragraph.

16. Line 250 was obtained instead of secured

Modified accordingly

17. Table 1 did authors collect information about household income ??

Not collected. We have collected the wealth index data

18. I think that the discussion needs a more in-depth, and authors have to present their major findings related to the population studied and not children aged between 6 to 23 months… to compare them to other studies conducted among lactating women (and explain in details the results obtained)
    Authors may add more statements in the end to highlight the implication and conclusions from this study

Thank you for the concerns, the major limitation for this research was shortage of literatures. We tried to amend according to the comment.
