# Supplementary material for: Adequate vitamin A rich food consumption and associated factors among lactating mothers visiting child immunization and post-natal clinic at health institutions in Gondar Town, Northwest Ethiopia
Source: PLoS One. 2020 Sep 21;15(9):e0239308. doi: 10.1371/journal.pone.0239308 (PMC7508326; doi:10.1371/journal.pone.0239308)
Supplement: Attachment — Submitted filename: Dear editor and reviewers.docx [file pone.0239308.s005.docx]

Dear Editor and Reviewer

Sincerest thanks for your response and reviewers comments on our manuscript. We have modified the paper in response to the extensive and insightful editor and reviewer comments. We would be glad to respond to any further questions and comments that you may have.

Response to Editor and Reviewer

1. I suggest to authors to add a paragraph about the HKI-FFQ as it is the main tool used in this paper at the Sampling method part (like it has been validated by WHO using serum retinol levels to provide reliable estimates of subclinical VAD.
   A brief description of the tool and then a paragraph about how authors have adapted the tool to local conditions ( what are questions that has been added or modified, steps )

- The HKI FFM asks respondents how many days in the last week consumed the foods listed on a predesigned FFQ. Only major sources of vitamin A are taken into consideration (t100 RE), though some attention are given to other foods. The tool has 28 different food item questions. From the 28 food items we used 21 food items which are available in the study area and consumed by the society, the remaining 7 food items like, noodles, Amaranth leaves, sweet potato leaves, cod liver oil, coconuts, weaning food fortified with vitamin A, Margarine fortified with vitamin A were not included in the tool because they are not commonly consumed in the study area.

2. Discussion needs some edits there is some suggestions :
   line 282 : our findings showed that the consumption of adequate vitamin A rich foods was….
   Line 283 : lactating women should have a slightly higher vitA requirements compared to women who are not pregnant or lactating as vitA in mother’s diet are directly transferred into breast milk.
   Line 287 : our results are higher than those found in Danot, Rural Kenya, ethiopia.. Indeed, in the Damot sore district, the study revealed that only 12,5% …..
   In rural kenya, only 13,5%.....
   While in India, the study revealed that …
   This difference in results can be explained by the effect of sample size, study settings , physiological state…..
   Please delete the time gap btw studies…
   Please delete paragraph 308…312 « no add value »
   Line 314 please modify unable to write and read by illiterate mothers (in all the manuscript)
   Line 316 : educational status by educational level
   Line 317 : because by may be explained by the fact that …
   Line 319 : a family size <= 3 had 4 times the most likely chance to…
   Line 320 : this may be due to a difficulty to provide….
   Please change “mothers may give…. By « mothers may sacrifice their own quality/quantity of nutrition in order to protect their children.
   Line 324 : this may be due to that mothers with low socioeconomic status may not have access or can afford to buy food rich in vitA…there is a strong and consistent evidence that food insecure mothers are at higher risk of malnutrition.
   Limitation
   Another limitation of this study is serum retinol levels were not determined , which could not be done ( limited budget, lack of required facilities….)

   Conclusion
   Line 347:
   Based on the HK threshold values, 61% of lactating women have inadequate vitA food consumption (not 38,9%) and thus, was not a public health problem among our population.
   Line 349: having college educational level and above,

- All the comments and suggestion has been addressed in the document.
