# Supplementary material for: Adequate vitamin A rich food consumption and associated factors among lactating mothers visiting child immunization and post-natal clinic at health institutions in Gondar Town, Northwest Ethiopia
Source: PLoS One. 2020 Sep 21;15(9):e0239308. doi: 10.1371/journal.pone.0239308 (PMC7508326; doi:10.1371/journal.pone.0239308)
Supplement: Attachment — Submitted filename: Response to editor and reviewers.docx [file pone.0239308.s006.docx]

Dear Editor and Reviewer

Sincerest thanks for your response and comments on our manuscript. We have modified the quality of English in this paper in response to the given comments. We would be glad to respond to any further questions and comments that you may have.
